# *Campylotropis xinfeniae* (Fabaceae, Papilionoideae), a new species from Yunnan, China, based on morphological and molecular evidence

**DOI:** 10.1002/ece3.11410

**Published:** 2024-05-20

**Authors:** Li‐Sha Jiang, Xin‐Hui Li, Xiong Li, Bo Xu

**Affiliations:** ^1^ CAS Key Laboratory of Mountain Ecological Restoration and Bioresource Utilization & Ecological Restoration and Biodiversity Conservation Key Laboratory of Sichuan Province, Chengdu Institute of Biology Chinese Academy of Sciences Chengdu China; ^2^ College of Pharmacy Guizhou University of Traditional Chinese Medicine Guiyang China; ^3^ College of Life Sciences University of Chinese Academy of Sciences Beijing China

**Keywords:** *Campylotropis*, chloroplast genome, dry‐hot valley, Leguminosae, Yunnan

## Abstract

*Campylotropis xinfeniae*, a new species from the dry‐hot valley of the Jinsha River in the Yunnan province, China, is described and illustrated. It is morphologically similar to *C. wilsonii* and *C. brevifolia* in having glabrescent old branches, absent stipels, 3‐foliolate leaves, and adaxially puberulent leaflets, while it differs from the latter two in having often paniculate inflorescences, obviously white standard, not incurved sickle keel, larger narrowly oblique legumes, and longer legume beak. The complete chloroplast genome of this new species is 149,073 bp in length and exhibits a typical quadripartite structure. Phylogenetic analyses based on the complete chloroplast genome also supported *C. xinfeniae* as a new species located at the basal distinct clade of the genus *Campylotropis*, clearly separated from the remaining members of the genus and its allied genera. A conservation assessment of data deficient (DD) is recommended for the new species without extensive exploring of similar habitats according to the IUCN Categories and Criteria.

## Graphical abstract

*Campylotropis xinfeniae*, a new species from the dry‐hot valley of the Jinsha River in the Yunnan province, China, is described and illustrated. Phylogenetic analyses based on the complete chloroplast genome supported *C. xinfeniae* as a new species located at the base of the genus *Campylotropis*.

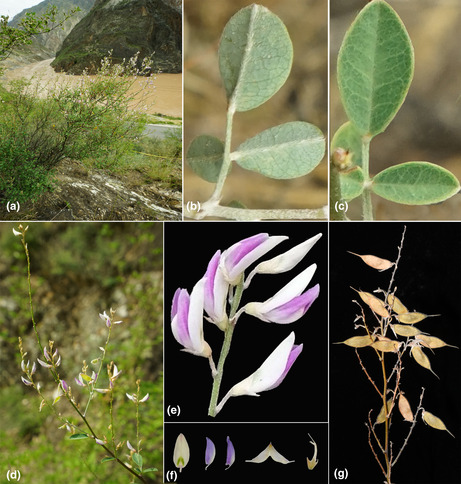

## 1 INTRODUCTION

*Campylotropis* Bunge (1835, p. 6) is a genus of the subtribe Lespedezinae in the tribe Desmodieae (Benth.) Hutch, which is considered one of the most advanced tribes within the largest subfamily Papilionoideae of the family Fabaceae (LWPG, 2017; Ohashi et al., 1981). Desmodieae has traditionally been divided into the subtribe Bryinae, the subtribe Desmodiinae, and the subtribe Lespedezinae (Ohashi et al., 1981). Subsequent studies placed the Bryinae in the Dalbergieae sensu lato, and the two genera *Phylacium* Benn. and *Neocollettia* Hemsl. were moved from the subtribe Lespedezinae to the tribe Phaseoleae (Bailey et al., 1997; Doyle et al., 2000; Kajita et al., 2001; Lavin et al., 2001). The tribe is now recognized as consisting of only two subtribes Lespedezinae and Desmodiinae, which correspond, respectively, to group *Lespedeza* and group *Desmodium* + *phyllodium* of the three groups further divided into the tribe (Ohashi, 2005). Molecular phylogenetic studies have strongly supported the monophyly of the Lespedezinae subtribe, including the three genera *Lespedeza* Michx., *Campylotropis* Bunge, and *Kummerowia* Schindl., and *Campylotropis* is related to *Kummerowia* and *Lespedeza* as sister groups (Han et al., 2010; Kajita et al., 2001; Nemoto et al., 2010; Stefanović et al., 2009).

This genus *Campylotropis* is characterized by one flower per subtending bract, usually caducous; pedicels articulate below the calyx; narrow and nearly sickle‐curved keel with a beak‐like acute apex (Fu, 1987; Huang, Ohashi, & Iowaka, 2010). *Campylotropis* is morphologically similar to *Lespedeza* and *Kummerowia* in usually absent stipels, legumes 1‐jointed, 1‐seeded, and not glochidiate, but it can easily be distinguished from *Lespedeza* by having one (vs. two in the latter) flower per subtending bract. *Campylotropis* can also easily be distinguished from *Kummerowia* by subulate stipules (vs. ovate stipules), arcuate lateral veins of leaflets (vs. strict lateral veins of leaflets), and not reaching to margin (vs. extending to margin) (Huang, Ohashi, Iokawa, & Nemoto, 2010; Iokawa & Ohashi, 2002). This genus comprises about 39 species and 11 infraspecific taxa, including the recently described *C. albopubescens* (Iokawa & H. Ohashi) M. Liao & B. Xu and *C. luquanensis* B. Xu & L.S. Jiang (Jiang & Xu, 2021; Liao & Xu, 2020), distributed in the temperate and tropical zones of Asia. China is the center of distribution of *Campylotropis*, boasting 32 species recognized, among which 20 species are endemic. Of these, 27 taxa including 12 endemics (with nine species, one subspecies, and two varieties) are found in Yunnan. (Fu, 1987; Huang, Ohashi, & Iowaka, 2010; Iokawa & Ohashi, 2002, 2008; Ohashi, 2005; Ohashi et al., 1981). However, the number of species in this region remains uncertain due to changing taxonomic status (Liao & Xu, 2020) and the continuous discovery of new species (Jiang & Xu, 2021). During the floristic investigation conducted in 2019 in the dry‐hot valley of the Three Parallel Rivers region in northwestern Yunnan Province, China, we encountered an unknown specimen belonging to the genus *Campylotropis*, which is different from any known species (Figure 3). This species may resemble *C. brevifolia* Ricker (1946, p. 37) and *C. wilsonii* Schindler (1912, p. 343) as they have glabrescent old branches, absent stipels, 3‐foliolate leaves, and adaxially puberulent leaflets, one flower per subtending bract (Huang, Ohashi, & Iowaka, 2010). However, the unknown specimens display smaller flowers with obvious white standard, paniculate inflorescences, and calyx tube longer than lobes, larger narrowly oblique legumes, and longer legume beak, which are distinguishable from the other two species (Table 1). We proposed the species new to science based on morphological characters and molecular phylogeny obtained from the chloroplast genome. This study confirmed and reported the discovery of this new species, *Campylotropis xinfeniae* B. Xu, X.H. Li & L.S. Jiang.

**TABLE 1:** Detailed comparison among *Campylotropis xinfeniae* and its morphologically related species.

Character	*C. xinfeniae*	*C. brevifolia*	*C. wilsonii*
Branchlet hair	Sparsely appressed short hairy	Densely white villous	Sparsely appressed short hairy
Stipule size	1–1.5 × 0.4–0.6 mm	2–2.5 × 0.8–1 mm	1.5–2.5 × 0.5–1 mm
Pinnate leave length	2–3.5 cm	1–2 cm	1.2–3.2 cm
Leave abaxial hairy	Sparsely appressed pubescent	Densely appressed white villous	Sparsely appressed pubescent
Inflorescence type	Often panicles	Racemes	Racemes
Inflorescence length	3.5–12 cm	1–2 cm	1–12 cm
Bracts and size	Persistent, 0.8–1 mm	Persistent, 1–1.5 mm	Caducous, 1–1.5 mm
Bracteoles and size	Persistent, 0.3–0.5 mm	Persistent, 0.5–1 mm	Caducous, 0.5–1 mm
Calyx length	Tube 1.7–2 mm, lobes 1–1.5 mm	Tube 1.7–2 mm, lobes 1.8–2 mm	Tube 1–1.2 mm, lobes 1–1.3 mm
Standard size	7.5–8.5 × 3.5–4.5 mm	8.5–9 × 4–5 mm	10.5–11 × 5–7 mm
Standard color	White	Reddish purple	Purple
Wing size	7–8 × 2–3 mm	9–9.5 × 3–3.5 mm	10–11 × 3–3.5 mm
Keel size	8–8.5 × 2–2.5 mm	10–12 × 3–3.5 mm	11–13 × 1.5–1.7 mm
Keel shape	Not incurved sickle	Incurved sickle	Incurved sickle
Pistil length	8.5–9.5 mm	10–11 mm	11–11.5 mm
Legume size	13–15 × 3.5–4 mm	8–9× 4–4.5 mm	10–12 × 3.5–4 mm
Legume shape	Narrowly oblique oblong	Obliquely obovate	Obliquely oblong
Legume beak length	3.5–4 mm	0.5–1 mm	2–2.5 mm

## 2 MATERIALS AND METHODS

### 2.1 Morphological analyses

Specimens from two populations of the new species were collected in Yunnan Province (Deqin County and Shangri‐La County). Morphological traits description and measurements (including the size, shape, color, and hair of the stems, leaves, flowers, and fruits; Table 1) of this new species were based on fresh field collections and dried herbarium specimens. The dried herbarium specimens representing the populations of Deqin County and Shangri‐La County were deposited in CDBI, GZTM, and PE (acronym of herbarium following Thiers, 2022). The morphological characters were measured by ImageJ v1.48 (Schneider et al., 2012), and the description followed the terminology in Beentje (2012) and Huang, Ohashi, and Iowaka (2010).

### 2.2 DNA extraction, sequencing, assembly and annotation

Fresh leaves of one representative individual of *Campylotropis xinfeniae* were collected and dried in silica gel as molecular studies (vouchers *Xin‐Hui Li linxinhui20190910*, deposited in the Herbarium CDBI). The total genomic DNA of *C. xinfeniae* was extracted using a modified cetyltrimethylammonium bromide (CTAB) method (Allen et al., 2006). The genomic DNA was fragmented, and library sizes were selected for 350 bp inserts. The paired‐end library was constructed and subsequently sequenced on BGI (the Beijing Genomics Institute, in Shenzhen, China) sequencing platform DNBseq‐T7. Approximately 21 Gb of raw data were generated for this new species. fastp v0.23.2 (Chen et al., 2018) was used to trim adapter‐containing and low‐quality reads from raw sequencing data. The complete chloroplast genome was assembled using GetOrganelle v1.7.5.3 (Jin et al., 2020). With reference to *C. brevifolia* (GenBank: OM775434), the chloroplast genome was annotated using PGA (Qu et al., 2019) and GeSeq (Tillich et al., 2017). Geneious Prime 8 (https://www.geneious.com) was used for manual adjustment of start and stop codons and the determination of pseudogenes. A circular map of the chloroplast genome was drawn using OGDRAW v1.3.1 (Greiner et al., 2019).

### 2.3 Phylogenetic analyses

To reveal the phylogenetic relationships of *Campylotropis xinfeniae* and its related species, this study included the newly generated sequence of *C. xinfeniae* and 22 accessions representing 17 *Campylotropis* species (including three subspecies, one variety, and one forma) in previous molecular phylogenetic analysis and five outgroups from GenBank (Table S1) (Feng et al., 2022; Jin et al., 2019; Le & Joonho, 2022; Oyebanji et al., 2020). The whole chloroplast genome sequences of all samples were aligned using MAFFT v7.508 (Katoh & Standley, 2013). The phylogenetic analyses were performed using Maximum likelihood (ML) and Bayesian inference (BI) methods based on whole chloroplast genomes. ModelFinder (Kalyaanamoorthy et al., 2017) was used to select the best‐fit model using the corrected Akaike Information Criterion (AICc). ML analysis was performed by IQ‐tree v2.1.4 (Nguyen et al., 2015) with a GTR + F + R4 model and 1000 bootstrap replicates. BI analysis was conducted using MrBayes v3.2.7a (Ronquist et al., 2012) with a TPM1uf + I + G model estimated by using jModelTest v2.1.10 (Darriba et al., 2012). The Markov Chain Monte Carlo algorithm was run to estimate the posterior probability (10 million generations and sampled every 1000 generations), with the preliminary 25% of sampled data discarded as burn‐in. The constructed phylogenetic trees were visualized using FigTree v1.4.4 (https://github.com/rambaut/figtree/releases/tag/v1.4.4).

## 3 RESULTS

### 3.1 Characteristics of the complete chloroplast genome

**FIGURE 1:**
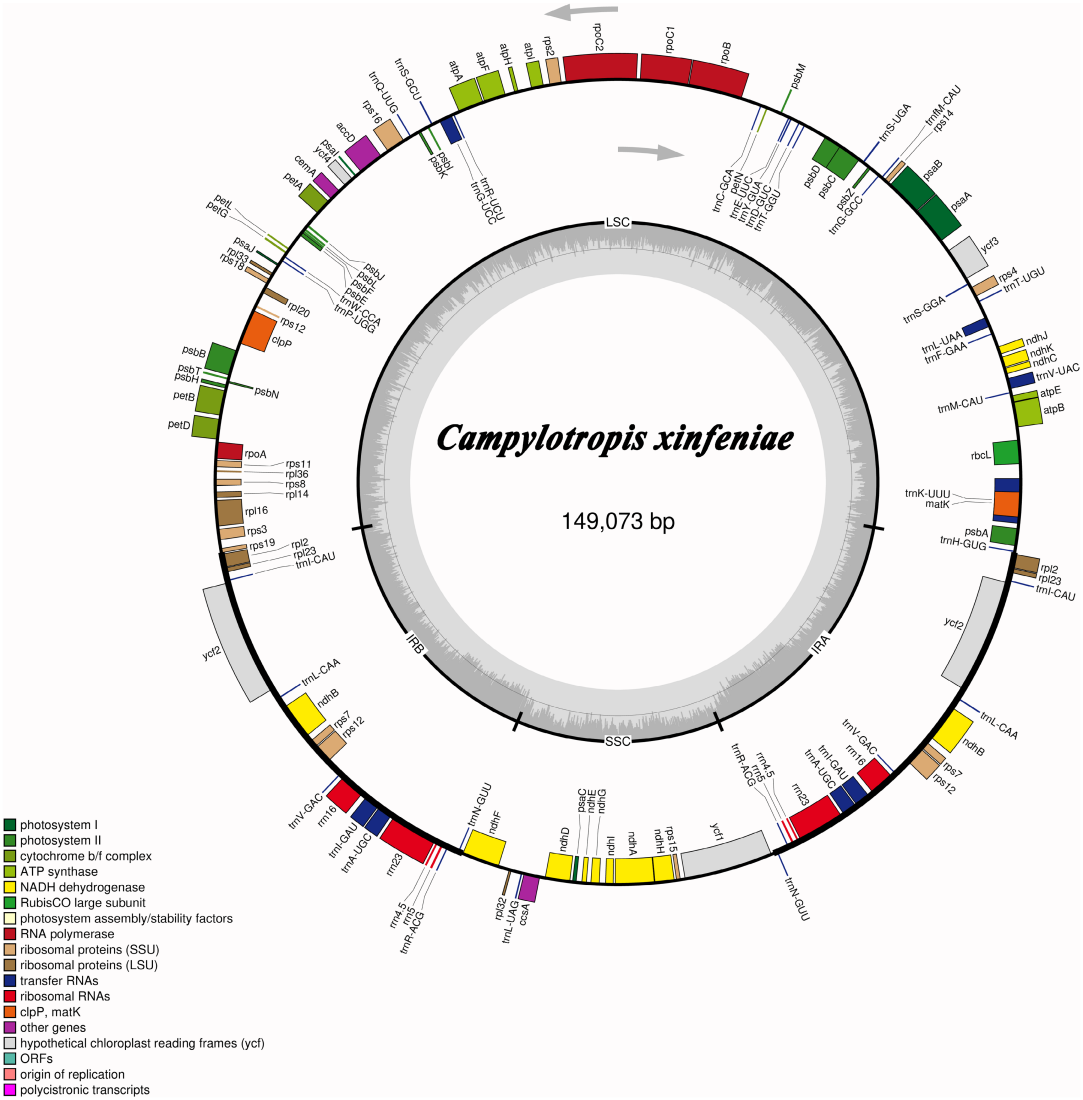
Chloroplast genome map of *Campylotropis xinfeniae*. The arrows indicate the transcription directions of the genes inside and outside of the circle. Genes belonging to different functional groups are color‐coded. The dark gray area in the inner circle denotes GC content, the light gray to the AT content of the genome. IR, inverted repeat; LSC, large single copy; SSC, small single copy.

The length of the chloroplast complete genome of *Campylotropis xinfeniae* sample was 149,073 bp (GenBank: OR506464, Figure 1). It possessed a typical quadripartite structure (LSC, SSC, IRa, and IRb). The characteristics and statistics of this new species chloroplast genome are summarized in Table 2.

**TABLE 2:** Basic characteristics of chloroplast genomes of *Campylotropis xinfeniae*.

Characteristic	*C. xinfeniae*
Total length (bp)	149,073
GC%	34.90%
LSC length (bp)	82,890
SSC length (bp)	18,821
IR length (bp)	23,681
Total genes	128
Protein‐coding genes	83
rRNA genes	8
tRNA genes	37

### 3.2 Phylogenetic relationship

**FIGURE 2:**
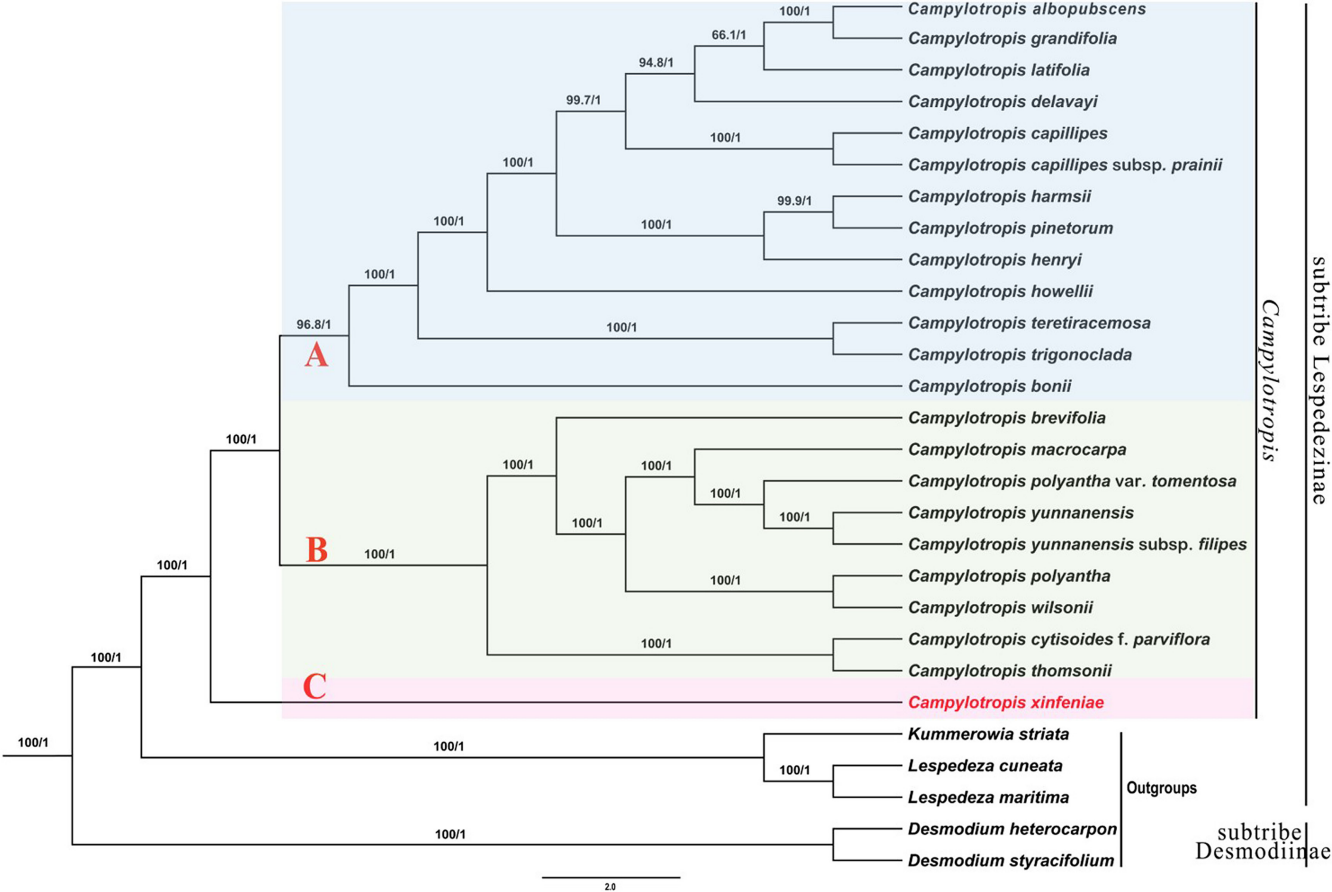
Phylogenetic relationships of *Campylotropis xinfeniae* and its related species in *Campylotropis*, inferred from Maximum Likelihood (ML) and Bayesian Inference (BI) methods, based on the whole chloroplast genomes. Numbers above branches indicate ML bootstrap supports (BS) and Bayesian posterior probabilities (PP). The phylogenetic position of *C. xinfeniae* is highlighted in red.

The phylogenetic trees inferred by ML and BI based on the whole chloroplast genome showed an identical topology and high support values (Figure 2). All topologies supported the mutual monophyly of the two subtribes Lespedezinae and Desmodiinae in tribe Desmodieae (100% bootstrap support [BS] and 1 posterior probability [PP], Figure 2). In the subtribe Lespedezinae, all phylogenetic analyses consistently supported the formation of a monophyletic group by the 23 taxa of *Campylotropis*. *Kummerowia striata* and the two *Lespedeza* species also formed a clade (BS = 100%, PP = 1, Figure 2). Phylogenetic analyses indicated that *Campylotropis* divided into two main clades: one with the putative new species *C. xinfeniae* (lineage C) as the basal group of this genus, sister to the remaining species, and the latter (BS = 100%, PP = 1) can be further subdivided into two subclades (lineages A and B) (Figure 2). Lineage A included *C. albopubescens*, *C. grandifolia*, *C. latifolia*, *C. delavayi*, *C. capillipes*, *C. capillipes* subsp. *prainii*, *C. pinetorum* subsp. *velutina*, *C. harmsii*, *C. henryi*, *C. howellii*, *C. teretiracemosa*, *C. trigonoclada*, and *C. bonii*. Lineage B included *C. brevifolia*, *C. macrocarpa*, *C. polyantha* var. *tomentosa*, *C. yunnanensis*, *C. yunnanensis* subsp. *filipes*, *C. polyantha*, *C. wilsonii*, *C. cytisoides* f. *parviflora*, and *C. thomsonii*. *Campylotropis xinfeniae* did not cluster into sister clades in the molecular phylogenetic trees with its two morphological relatives, *C. brevifolia* and *C. wilsonii*, which are genetically difference despite being morphologically similar.

## 4 TAXONOMIC TREATMENT

**FIGURE 3:**
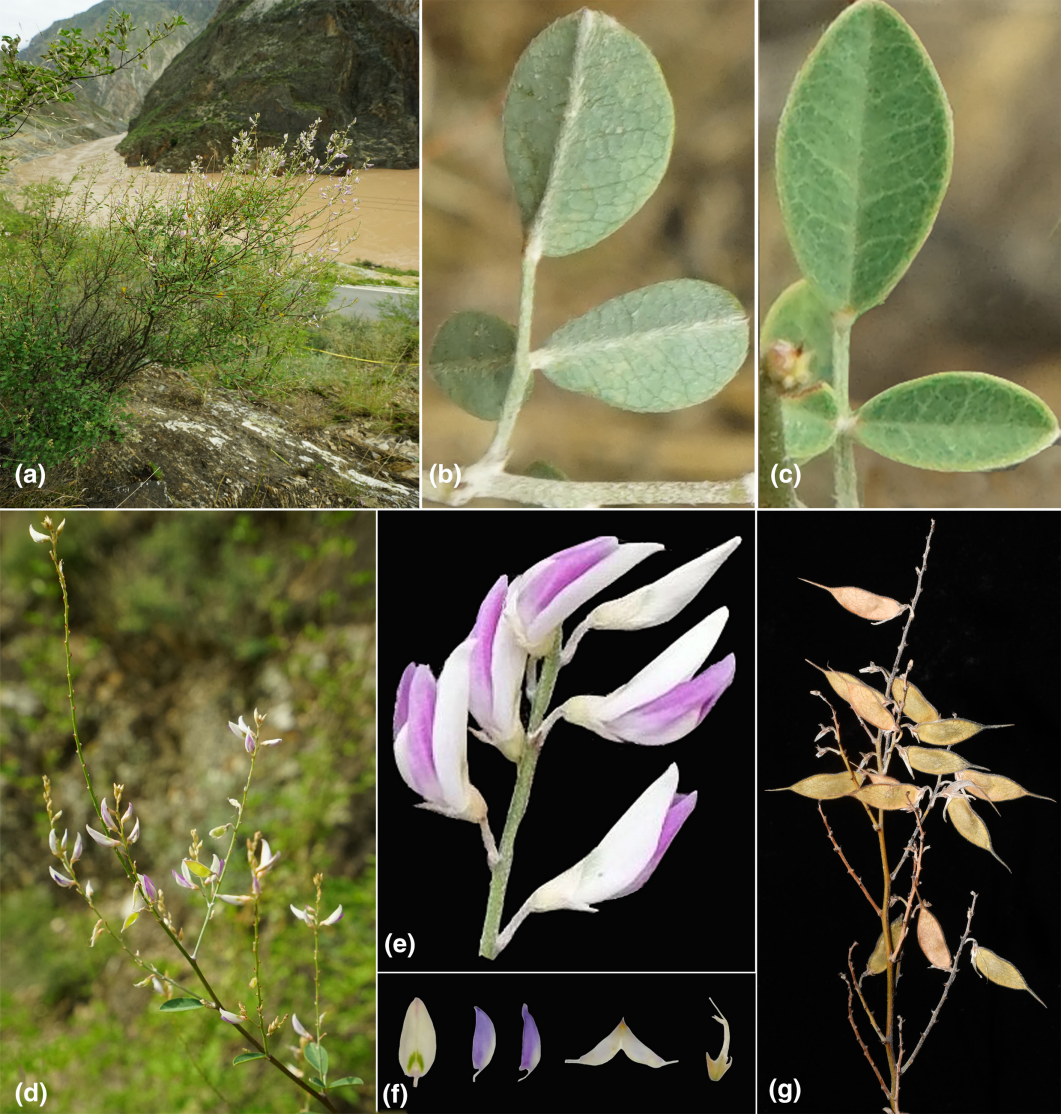
*Campylotropis xinfeniae*. (a) Habitat; (b) Leaves (abaxial); (c) Leaves (adaxial); (d) Branch and inflorescence; (e) Raceme, showing rachis, peduncle, and pedicels; (f) Detail of flower, standard, wings, keels, calyx, stamens and pistil; (g) Infructescence and legumes.

**FIGURE 4:**
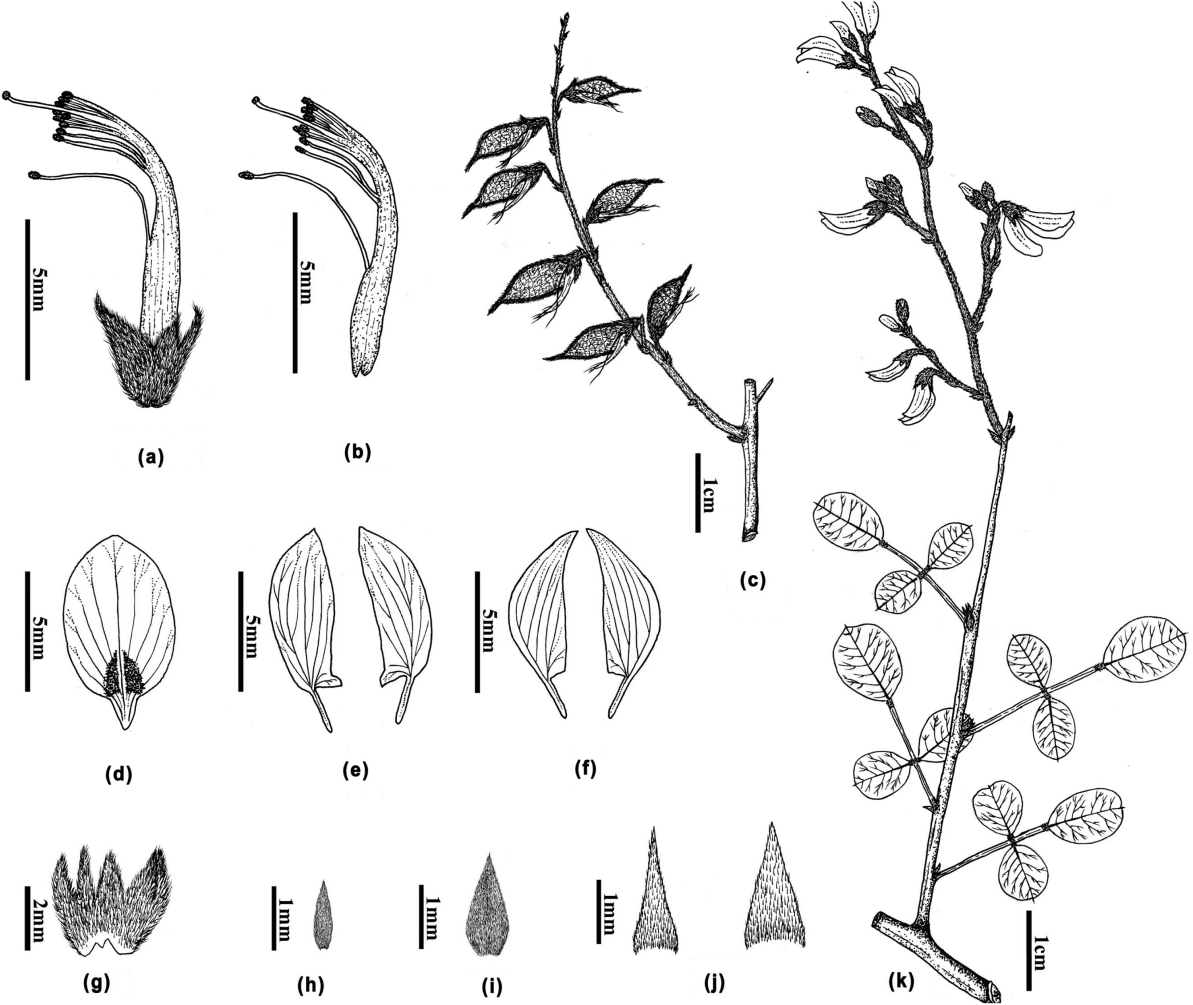
Illustration of *Campylotropis xinfeniae*. (a) & (b) Stamens, pistils, and calyx; (c) Fruit branch; (d) Standard; (e) Wings; (f) Keels; (g) Calyx; (h) Bracteole; (i) Bract; (j) Stipules; (k) Branch. Drawn by Mr. Yin‐Zhang Wang based on the holotype (and (c) is also drawn in reference to the isotypes).

***Campylotropis xinfeniae*** B. Xu, X.H. Li & L.S. Jiang, *sp. nov*. (Figures 3 and 4).

### 4.1 Type

CHINA. Yunnan: Diqing Tibetan Autonomous Prefecture, Deqin County, 28.38373° N, 99.19025° E, elev. 2400–2500 m, growing on rocky slopes, 10 September 2019, *X. H. Li linxinhui20190910* (holotype: CDBI barcode CDBI0293333!; isotypes: CDBI barcodes CDBI0293334!, CDBI0293335!).

### 4.2 Diagnosis

This new species *Campylotropis xinfeniae* is morphologically similar to *C. brevifolia* and *C. wilsonii* in having glabrescent old branches, absent stipels, 3‐foliolate leaves, and adaxially glabrous or sparse puberulent leaflets, one flower per subtending bract, but it differs from both in having often paniculate inflorescences (vs. racemes and racemes), obviously white standard (vs. reddish purple and purple), not incurved sickle keel (vs. incurved sickle and incurved sickle), shorter pistil (8.5–9.5 mm vs 10–11 mm and 11–11.5 mm), larger oblique legumes (13–15 × 3.5–4 mm vs. 8–9× 4–4.5 mm and 10–12 × 3.5–4 mm), and longer legume beak (3.5–4 mm vs. 0.5–1 mm and 2–2.5 mm).

### 4.3 Description

Shrubs or shrublets, erect, 0.4–2 m tall. Branches glabrescent, young branchlets sparsely appressed short hairy. Leaves pinnately 3‐foliolate, 2–3.5 cm long; stipules triangular‐lanceolate to lanceolate, 1–1.5 (−2) mm long, 0.4–0.6 mm wide; stipels absent; petioles sparsely appressed short hairy, 0.6–1.5 cm long; leaflets widely elliptic and widely obovate, rarely elliptic and ovate, terminal one 0.8–1.6 cm long, 0.8–1.3 cm wide, abaxially usually sparsely appressed pubescent, adaxially glabrous or sparsely puberulent, midveins distinctly raised, apex retuse and mucronate, rarely obtuse to rounded, base suborbicular, rarely rounded and broadly cuneate. Inflorescences 3.5–12 cm long, often axillary and terminal paniculate, rarely axillary racemose, peduncles (0.5–) 1–3 cm long, densely appressed pubescent; pedicels 1.5–3 mm long, rachis and pedicels densely appressed pubescent. Bracts persistent, ovate‐lanceolate or lanceolate, 0.8–1 mm long, bracteoles usually persistent, narrowly triangular‐lanceolate, 0.3–0.5 mm long. Calyx campanulate, 2.5–3.5 mm long, with dense appressed short hairs, tube 1.5–2 mm long; lobes narrowly triangular and acuminate, 1–1.5 mm long, 0.6–0.7 (−1.2) mm wide, calyx tube longer than lobes, lower lobes longer than upper and lateral ones, upper lobes connate. Corolla pale purple; standard white, elliptic to ovate, rarely suborbicular, 7.5–8.5 mm long, 3.5–4.5 mm wide, apex obtuse, claw 1–1.3 mm long; wings pale purple, auriculate, 7–8 mm long, 2–3 mm wide, apex acute, auricle ca. 1 mm long, claw ca. 2 mm long; keel white, not incurved sickle, 8–8.5 mm long, 2–2.5 mm wide, claw 2–2.3 mm long, auricle ca. 0.5 mm long. Stamens diadelphous, 8–8.5 mm long, staminal tubes 6–6.5 mm long, free part of filaments 2–3 mm long. Pistils 8.5–9.5 mm long; ovary sparse puberulent, upper margin with densely ciliate; style incurved, 5–6 mm long. Legume narrowly oblique elliptic, light brown, and sparsely pubescent, 13–15 mm long, 3.5–4 mm wide; apex beaked, 3.5–4 mm long. Seeds reniform, 4–5 mm long, 2.5–3 mm wide.

### 4.4 Phenology

Flowering and fruiting from July to October.

### 4.5 Etymology

The specific epithet honors Professor Xin‐Fen Gao, an outstanding botanist based at Chengdu Institute of Biology, Chinese Academy of Sciences, for her contributions to the taxonomy of *Campylotropis* (Gao, 2006; Gao et al., 1992). Its Chinese name, xìn fēn háng zǐ shāo (信芬杭子梢), is also suggested here.

### 4.6 Distribution and habitat

**FIGURE 5:**
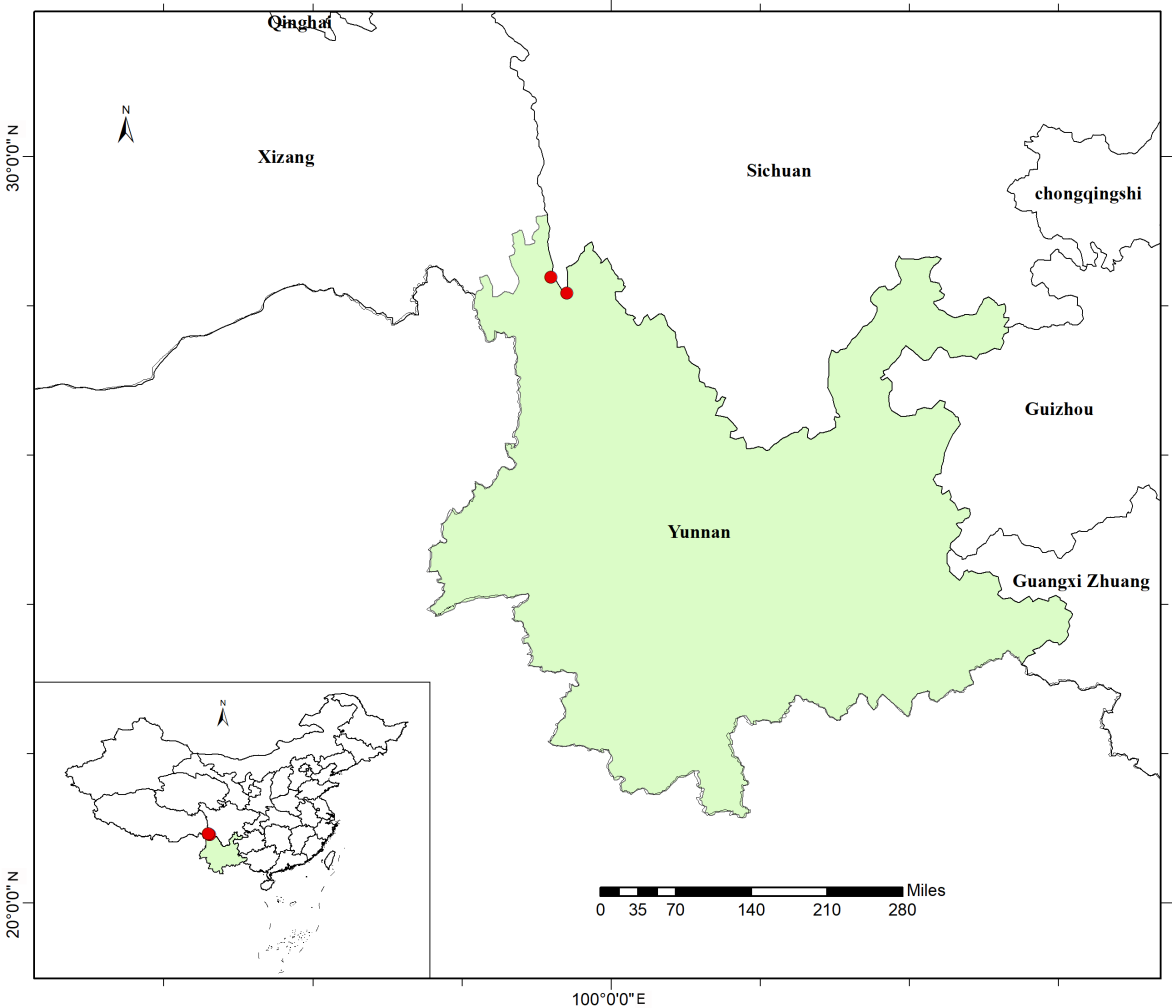
Distribution map of *Campylotropis xinfeniae* (red circles).

This species so far was only from the dry‐hot valley on the Jinsha River in the Yunnan Province (Figure 5). It was observed to grow rocky slopes in dry‐hot valleys at elevations of 2100–2500 m.

### 4.7 Additional specimens examined

CHINA. Yunnan, Diqing Tibetan Autonomous Prefecture, Shangri‐La County, Nixi Township, Shangqiaotou Village, elev. ca. 2100 m, 2 July 1981, *Qinghai‐Tibet Team 00183826* (PE); Deqin County, elev. 2400–2500 m, 21 October 2019, *X. H. Li 2907* (CDBI! GZTM!) (This specimen and type specimen were collected from the same location at different times).

### 4.8 Conservation status

*Campylotropis xinfeniae* is only known from two counties of Yunnan Province, located in the Jinsha River valley zone of the Hengduan Mountains, where sparse vegetation exists in a dry and warm river valley, with high temperatures and minimal precipitation due to the foehn effect. The new species grows in the gallery or riverbank along the dry‐hot valley of the upper reaches of the Jinsha River that we investigated (dominant by *Sophora davidii* Kom. ex Pavol.), mainly on alkaline brown soil (Chang & Li, 2022; Du et al., 2022; Zhang et al., 2013). It is accompanied by *Bauhinia rufescens* Lam., *Indigofera silvestrii* Pamp., *Stemona japonica* (Blume) Miq., *Incarvillea arguta* (Royle) Royle, *Rosa soulieana* Crép., and unidentified species of Gramineae. Due to the absence of further exploration in adjacent similar habitats at present, the assessment is therefore categorized as data deficient (DD) according to the IUCN Red List Categories and Criteria (IUCN Standards and Petitions Committee, 2022).

### 4.9 Taxonomic note

Morphologically, the new species is morphologically similar to *C. brevifolia* and *C. wilsonii* by sharing a series of similar features, including glabrescent old branches, absent stipels, 3‐foliolate leaves, and adaxially glabrous or sparsely puberulent leaflets, one flower per subtending bract (Huang, Ohashi, Iokawa, & Nemoto, 2010; Huang, Ohashi, & Iowaka, 2010; Iokawa & Ohashi, 2002). However, *C. xinfeniae* can be easily distinguished from *C. brevifolia* due to its sparsely appressed short hairy branchlet (vs. densely white villous), smaller stipules (1–1.5 × 0.4–0.6 mm vs. 2–2.5 × 0.8–1 mm), longer pinnate leaves (2–3.5 cm vs. 1–2 cm), abaxially sparsely appressed pubescent (vs. densely appressed white villous), longer paniculate inflorescences (3.5–12 cm vs. 1–2 cm, panicles vs. racemes), white standard (vs. reddish purple), not incurved sickle keel (vs. incurved sickle), larger narrowly oblique oblong legumes (13–15 × 3.5–4 mm vs. 8–9 × 4–4.5 mm, oblong vs. obovate), and longer legume beak (3.5–4 mm vs. 0.5–1 mm). In addition, *C. xinfeniae* can be easily distinguished from *C. wilsonii* by smaller stipules (1–1.5 × 0.4–0.6 mm vs. 1.5–2.5 × 0.5–1 mm), often panicles (vs. racemes), smaller persistent bracts (0.8–1 mm vs. 1–1.5 mm, persistent vs. caducous), smaller persistent bracteoles (0.3–0.5 mm vs. 0.5–1 mm, persistent vs. caducous), white standard (vs. purple), not incurved sickle keel (vs. incurved sickle), larger legumes (13–15 × 3.5–4 mm vs. 10–12 × 3.5–4 mm), and longer legume beak (3.5–4 mm vs. 2–2.5 mm). Detailed morphological comparison among the three species is summarized in Table 1.

Phylogenetically, the monophyly of the two subtribes within Desmodieae was obtained from the phylogenetic analysis in this study had been previously confirmed in previous research (Jabbour et al., 2018; Jin et al., 2019). Similarly, the subtribe Lespedezinae also included three genera: *Campylotropis*, *Lespedeza*, and *Kummerowia* (Han et al., 2010; Kajita et al., 2001; Nemoto et al., 2010; Stefanović et al., 2009, Figure 2). In this study, the sister relationship between *Lespedeza* and *Kummerowia* was consistent with the findings based on molecular markers in phylogenetic studies (Jabbour et al., 2018; Xu et al., 2012). Additionally, the sister relationship between the two genera and *Campylotropis* in this study was reaffirmed and confirmed (Jin et al., 2019). The phylogenetic analysis indicated that *Campylotropis* is monophyletic for the groups considered (BS = 100%, PP = 1, Figure 2), which is consistent with the results of Feng et al. (2022) based on the whole chloroplast genome. However, there are differences in the intrageneric and interspecific relationships of *Campylotropis*. For instance, the position of *C. bonii* is no longer a distinct lineage in this study, and the relatively low support between sister lineages in Feng et al. (2022), left the phylogenetic relationships unresolved. The genus can also be divided into three lineages (Figure 2). Lineage C contained only one species, *C. xinfeniae* was a sister to all the remaining species of *Campylotropis* (lineage A and lineage B). Lineage A was mostly restricted to southwestern China and Southeast Asia, and lineage B contained regional endemic (e.g., *C. wilsonii*) and widely distributed (e.g., *C. macrocarpa*) species (Huang, Ohashi, & Iowaka, 2010). The phylogenetic analysis revealed that *C. xinfeniae* is a distinct new species within the *Campylotropis* genus, positioned at the basal clade separate from other members and related genera. (BS = 100%, PP = 1, Figure 2). *Campylotropis xinfeniae* did not form sister clades with its two morphological relatives, *C. brevifolia* and *C. wilsonii*, in the molecular phylogenetic trees (BS = 100%, PP = 1, Figure 2). Although they shared morphological similarities, there were distinct genetic differences between them. Additionally, it can be differentiated morphologically by often paniculate inflorescences, obviously white standard, not incurved sickle keel, larger narrowly oblique elliptic legumes, and longer legume beak.

## AUTHOR CONTRIBUTIONS

**Li‐Sha Jiang:** Formal analysis (equal); methodology (lead); writing – original draft (lead); writing – review and editing (equal). **Xin‐Hui Li:** Investigation (lead). **Xiong Li:** Formal analysis (equal); methodology (supporting). **Bo Xu:** Funding acquisition (lead); project administration (lead); writing – review and editing (equal).

## FUNDING INFORMATION

The National Natural Science Foundation of China, Grant/Award Numbers: 31560063 & 31860126.

## CONFLICT OF INTEREST STATEMENT

There is no conflict of interest to declare.

## Supporting information


**TABLE S1.** GenBank Accession for 22 taxa in *Campylotropis* and outgroups used in this study.

## Data Availability

The sequences of this study have been deposited in The National Center for Biotechnology Information (NCBI) database (https://www.ncbi.nlm.nih.gov). The whole chloroplast genome analyzed for this study can be found in the GenBank with accession number OR506464. Appendix Table to this article can be found online.
