# Supplementary material for: Campylotropis xinfeniae (Fabaceae, Papilionoideae), a new species from Yunnan, China, based on morphological and molecular evidence
Source: Ecol Evol. 2024 May 20;14(5):e11410. doi: 10.1002/ece3.11410 (PMC11103638; doi:10.1002/ece3.11410)
Supplement: Supplementary file 1 — TABLE S1. GenBank Accession for 22 taxa in Campylotropis and outgroups used in this study. [file ECE3-14-e11410-s001.docx]

**Table S1.** GenBank Accession for 22 taxa in *Campylotropis* and outgroups used in this study.

| **Species name** | **GenBank Accession** |
| --- | --- |
| *C. albopubescens* | OM775444 |
| *C. bonii* | OM775455 |
| *C. brevifolia* | OM775434 |
| *C. capillipes* | OM775435 |
| *C. delavayi* | OM775436 |
| *C. grandifolia* | OM775437 |
| *C. harmsii* | OM775438 |
| *C. henryi* | OM775439 |
| *C. howellii* | OM775440 |
| *C. latifolia* | OM775441 |
| *C. macrocarpa* | NC_044100 |
| *C. cytisoides f. parviflora* | OM775442 |
| *C. pinetorum subsp. velutina* | OM775443 |
| *C. polyantha* | OM775447 |
| *C. polyantha var. tomentosa* | OM775445 |
| *C. capillipes subsp. prainii* | OM775446 |
| *C. teretiracemosa* | OM775449 |
| *C. thomsonii* | OM775450 |
| *C. trigonoclada* | OM775451 |
| *C. wilsonii* | OM775452 |
| *C. yunnanensis subsp. filipes* | OM775453 |
| *C. yunnanensis* | OM775454 |
| *Desmodium heterocarpon* | MG867567 |
| *Desmodium styracifolium* | MN913536 |
| *Kummerowia striata* | MG867569.1 |
| *Lespedeza cuneata* | MN966635.1 |
| *Lespedeza maritima* | MG867570.1 |
